# Evaluation of trimetazidine in alleviating paclitaxel-induced peripheral neuropathy in breast cancer patients: a randomized controlled trial

**DOI:** 10.3389/fphar.2026.1748399

**Published:** 2026-02-02

**Authors:** Asmaa N. Iqbal, Fatma M. Mady, Eman Mohamed Sadek, Noha M. Abdullah, Ahmad Mostafa Abdel-Azeez, Eman Shorog, Engy A. Wahsh

**Affiliations:** 1 Clinical Pharmacy Department, Faculty of Pharmacy, Minia University, Minya, Egypt; 2 Pharmaceutics Department, Faculty of Pharmacy, Minia University, Minya, Egypt; 3 Department of Clinical Pathology, Faculty of Medicine, Minia University, Minya, Egypt; 4 Department of Clinical Oncology, Faculty of Medicine, Minia University, Minya, Egypt; 5 Department of Clinical Pharmacy, College of Pharmacy, King Khalid University, Abha, Saudi Arabia; 6 Clinical Pharmacy Department, Faculty of Pharmacy, October 6 University, Giza, Egypt

**Keywords:** breast cancer, nerve growth factor, peripheral neuropathy, quality of life, trimetazidine, paclitaxel-induced neuropathy

## Abstract

**Background:**

Paclitaxel-induced peripheral neuropathy is a frequent chemotherapy complication that causes nerve damage and profoundly reduces patients’ quality of life. Despite extensive preclinical evidence supporting the neuroprotective potential of trimetazidine against peripheral neuropathy, its clinical efficacy remains unexplored.

**Objectives:**

This proof-of-concept randomized controlled trial aimed to investigate the effect of trimetazidine administered during the early phase of treatment on the incidence of paclitaxel-induced peripheral neuropathy in patients with non-metastatic breast cancer.

**Methods:**

This parallel randomized placebo-controlled blinded endpoint trial was conducted at the Oncology Center, Minia University, Egypt, involving 60 breast cancer patients scheduled to receive weekly paclitaxel 90 mg/m^2^. Patients were randomized to receive either trimetazidine 35 mg once daily or placebo alongside standard care. Measurements included the incidence of paclitaxel-induced neuropathy assessed by the National Cancer Institute’s Common Terminology Criteria for Adverse Events (NCI-CTCAE) version 5.0, patient quality of life via the Functional Assessment of Cancer Therapy/Gynecologic Oncology Group–Neurotoxicity (FACT-GOG-Ntx) subscale, and exploratory serum biomarkers, specifically nerve growth factor (NGF) levels. Neuropathy and biomarkers were evaluated over an 8-week period.

**Main Results:**

The incidence of grade 2 and 3 peripheral neuropathies was significantly lower in the trimetazidine group compared to controls, with notable reductions in paresthesia (p = 0.037), peripheral motor neuropathy (p = 0.004), and dysesthesia (p = 0.045), except for peripheral sensory neuropathy (p = 0.152). Clinically significant worsening in neuropathy-related quality of life was more frequent in the control group compared to the trimetazidine group (p = 0.001). Additionally, the trimetazidine group exhibited a significantly greater percentage increase in serum nerve growth factor from baseline (p = 0.003).

**Conclusion:**

Trimetazidine offers a safe and effective option for mitigating early paclitaxel-induced peripheral neuropathy in breast cancer patients. Further large-scale studies with longer follow-up are warranted to confirm these findings and explore effects across different chemotherapy regimens.

**Clinical Trial Registration:**

https://clinicaltrials.gov/study/NCT06459193, identifier NCT06459193.

## 1 Introduction

Chemotherapy-induced peripheral neuropathy (CIPN) is a common and debilitating complication affecting 60%–90% of patients receiving neurotoxic agents like taxanes and platinum compounds. CIPN manifests as sensory symptoms—numbness, tingling, burning—in a stocking-glove distribution, significantly impairing quality of life and often leading to chemotherapy dose reductions or discontinuation, thereby compromising oncologic outcomes (Tanabe et al., 2013; Chan et al., 2019; Ashok Kumar et al., 2025; Bacalhau et al., 2023). In breast cancer patients, CIPN prevalence reaches 72.9% at chemotherapy completion, with around 31% experiencing persistent symptoms months later (Gehr et al., 2025).

Breast cancer is the most frequently diagnosed cancer in women globally, with approximately 2.3 million new cases and 670,000 deaths reported in 2022. Incidence varies geographically, exceeding 100 per 100,000 women in high-risk regions such as Australia and Northern Europe. Standard systemic treatment of breast cancer combines chemotherapy, endocrine therapy for hormone receptor–positive disease, and HER2-targeted agents for HER2-positive tumors, in addition to surgery and radiotherapy. Taxanes, particularly paclitaxel and docetaxel, are key components of adjuvant, neoadjuvant, and metastatic regimens for patients at higher risk of recurrence. Large trials and meta-analyses have shown that adding a taxane to anthracycline-based chemotherapy reduces recurrence and improves overall survival in early breast cancer, which has established taxane-based combinations such as anthracycline followed by paclitaxel (AC→T) as standard of care (Kim et al., 2025; World Health Organization WHO, 2024; Kawashiri et al., 2021). However, paclitaxel-induced peripheral neuropathy, a dose-limiting toxicity, significantly challenges treatment adherence and patient quality of life, especially amid a projected 38% worldwide rise in breast cancer incidence by 2050 (Ashok Kumar et al., 2025; Kim et al., 2025; Timmins et al., 2021).

Paclitaxel-induced peripheral neuropathy (PIPN) affects 60%–80% of breast cancer patients during or soon after treatment, with many experiencing chronic symptoms (Chan et al., 2019; Lixian et al., 2024). Onset and severity correlate with cumulative dose, typically emerging after ∼250–275 mg/m^2^ (around 1.7 cycles) and worsening near 400–540 mg/m^2^. Approximately 25% of patients require dose adjustments due to severe neuropathy (Timmins et al., 2021; Rowinsky and Donehower, 1995). Mechanistically, PIPN results from paclitaxel-induced microtubule stabilization disrupting axonal transport, mitochondrial dysfunction, oxidative stress, and neuroinflammation involving TLR4/p38/NF-κB pathways and pro-inflammatory cytokines (IL-1β, TNF-α). Complement activation and mitochondrial trafficking defects further exacerbate nerve injury (Bacalhau et al., 2023; Miaskowski et al., 2019; Cristiano et al., 2023; Hammad A. S. et al., 2023). Individual risk factors include age, baseline inflammation, and cumulative dose (Ghoreishi et al., 2018; Hiramoto et al., 2021). Despite the heavy clinical burden, treatment options are limited. Current approaches to managing paclitaxel-induced peripheral neuropathy in breast cancer mainly focus on symptomatic relief and chemotherapy modification rather than true neuroprotection. Cryotherapy provides modest relief, while duloxetine, currently the only pharmacologic agent recommended by the American Society of Clinical Oncology (ASCO) for painful CIPN, offers limited efficacy and may have tolerability issues. Several other pharmacological strategies—including gabapentinoids, tricyclic antidepressants, venlafaxine, topical compounded creams, and antioxidant or neuroprotective agents such as glutathione, acetyl L carnitine, vitamin E, calcium–magnesium infusions, and cilostazol—have been assessed in clinical trials, but results have been inconsistent and none are currently recommended for routine prevention of PIPN (Kawashiri et al., 2021; Lu et al., 2020; Dewidar et al., 2025; Haroun et al., 2023; Khalefa et al., 2020).

Trimetazidine, a metabolic modulator with established cardioprotective benefits, is increasingly recognized for its neuroprotective potential in oncology. By shifting cellular metabolism from fatty acid β-oxidation to glucose oxidation, trimetazidine improves ATP production and reduces oxidative stress and calcium overload, protecting cardiomyocytes and potentially peripheral neurons (Dézsi, 2016; Abdeljalil et al., 2023). It also attenuates neuroinflammation by downregulating key pathways such as TLR4/p38/NF-κB and enhances neuronal survival via upregulation of proteins like klotho and progranulin (Hammad A. S. et al., 2023; Nasser et al., 2022). Importantly, preclinical models demonstrate its ability to synergize with chemotherapies like doxorubicin and abraxane without impairing anticancer efficacy, thereby reducing chemotherapy toxicity (Abdeljalil et al., 2023; Atlı Şekeroğlu et al., 2022). Clinically, trimetazidine reduces cardiotoxicity in breast cancer patients receiving anthracyclines without affecting treatment outcomes (Delle Donne et al., 2020; Sadigova, 2023). Administered as a modified-release 35 mg once daily tablet, trimetazidine offers convenient dosing with sustained plasma levels, comparable to regimens effective in neuroinflammatory disorders such as fibromyalgia (Ahmad, 2010). Given that PIPN is driven by mitochondrial dysfunction, oxidative stress, and neuroinflammation, these metabolic and anti-inflammatory actions provide a strong biological rationale for investigating trimetazidine as a preventive strategy for paclitaxel induced peripheral neuropathy in breast cancer patients.

Nerve Growth Factor (NGF) is vital for the survival, growth, and function of peripheral sensory neurons and modulates pain signaling and neuroinflammation. In CIPN, NGF levels decline as neuropathy develops and worsen, correlating with neurological deficits and nerve damage severity. Patients with CIPN exhibit significantly reduced circulating NGF compared to those without neuropathy, indicative of sensory neuron degeneration (Youk et al., 2017; Irsyad et al., 2025; Chan et al., 2004; Chang et al., 2016). This NGF decrease distinguishes CIPN from other neuropathic pain states where NGF may rise. Trimetazidine’s modulation of NGF signaling underscores NGF’s relevance as a biomarker for evaluating its therapeutic potential in mitigating CIPN progression and symptoms.

These findings support trimetazidine as a promising therapy to prevent or reduce early PIPN in breast cancer patients, addressing the critical need for safe, effective neuroprotection to improve chemotherapy tolerance and quality of life. Notably, no clinical trials have yet assessed trimetazidine’s preventive role in PIPN, underscoring this study’s novelty and significance (Kawashiri et al., 2021; Hammad A. S. et al., 2023).

This exploratory randomized controlled trial evaluates trimetazidine’s efficacy and safety in reducing early PIPN in breast cancer patients. It assesses trimetazidine’s effects on neuropathic symptoms, nerve biomarker, and quality of life during the first 8 weeks of chemotherapy, aiming to improve treatment tolerability and outcomes.

## 2 Methods

### 2.1 Study design

This study was conducted as a randomized, single-blind, placebo-controlled, parallel-group clinical trial. The study protocol was approved by the Research Ethics Committee at the Faculty of Pharmacy, Minia University (approval number: MPEC 2301006, ClinicalTrials.gov ID: NCT06459193). All procedures were strictly conducted following the ethical standards set forth in the Declaration of Helsinki. Written informed consent was obtained from all participants prior to their inclusion. Patient enrollment spanned from June 2024 to January 2025, with the follow-up phase concluding in March 2025.

### 2.2 Patient population and chemotherapy protocol

Participants were consecutively recruited from the Oncology Center at Minia University. Eligibility criteria included female patients diagnosed with breast cancer, scheduled to undergo paclitaxel chemotherapy, and possessing an Eastern Cooperative Oncology Group (ECOG) performance status between 0 and 2. Adequate bone marrow function was required, defined by a white blood cell count ≥4,000/mm^3^ and platelet count ≥100,000/mm^3^. At baseline, all participants exhibited satisfactory hepatic, renal, and hematologic function, evidenced by normal values of alanine transaminase (ALT), aspartate transaminase (AST), serum creatinine, blood urea nitrogen, platelet count, and lymphocyte count.

Participants were excluded if they showed any neuropathy symptoms at baseline; had diagnoses of diabetes mellitus, alcoholic liver disease, or heart failure; were pregnant or breastfeeding; or were taking vitamins or supplements that might interfere with the study. Additionally, those with contraindications to trimetazidine—such as Parkinson’s disease, related movement disorders, tremors, or restless leg syndrome—were not eligible for inclusion.

All participants completed four cycles of adjuvant AC chemotherapy with doxorubicin (60 mg/m^2^) and cyclophosphamide (600 mg/m^2^), followed by planned intravenous paclitaxel administration at 90 mg/m^2^. Although the standard weekly paclitaxel regimen extends over 12 weeks, this study’s follow-up was limited to the initial 8 weeks to capture the early onset and progression phase of PIPN.

#### 2.2.1 Premedication and antiemetic regimen

Patients received standard premedication with dexamethasone, with or without the antiemetic ondansetron, before each chemotherapy cycle, following clinical guidelines to prevent hypersensitivity reactions and chemotherapy-induced nausea and vomiting, ensuring safety and tolerability.

### 2.3 Randomization and study interventions

The trial was conducted as a randomized, placebo-controlled, single-blind study. Due to the nature of the intervention, participants were aware of their assigned treatment groups, while the outcome assessor remained blinded to minimize bias. The inclusion of a placebo control accounted for psychological and procedural effects potentially impacting outcomes. Randomization was carried out using a computer-generated sequence created with Random Allocation Software, with allocation concealment maintained through sequentially numbered, opaque envelopes opened only at the time of intervention administration. The control arm received placebo tablets in conjunction with standard paclitaxel chemotherapy, whereas the intervention arm received paclitaxel combined with trimetazidine 35 mg once daily. The trimetazidine dosage was selected based on expert clinical opinion and prior evidence demonstrating its safety and efficacy at this dose in fibromyalgia and related conditions (Ahmad, 2010). Placebo tablets, consisting of starch and visually identical to active trimetazidine tablets, were administered to control participants. Subjects were instructed to take the study medication during dinner. The study intervention commenced 1 week before the initiation of paclitaxel treatment and was maintained until the completion of the eighth week.

### 2.4 Efficacy outcomes

#### 2.4.1 Primary outcomes

The primary endpoints included the incidence and severity of neurologic adverse events, evaluated weekly by clinicians using the National Cancer Institute’s Common Terminology Criteria for Adverse Events (NCI-CTCAE) version 5.0 (National Cancer Institute, 2017). The assessed conditions were:

- Peripheral sensory neuropathy: impairment or dysfunction of peripheral sensory nerves.
- Peripheral motor neuropathy: damage or dysfunction of peripheral motor nerves.
- Dysesthesia: altered sensory perception manifesting as abnormal and unpleasant sensations.
- Paresthesia: abnormal cutaneous sensations such as tingling, numbness, pressure, cold, and warmth caused by sensory neuron dysfunction.

The primary measure was the difference between groups in the occurrence of these adverse events rated grade 2 or above. Each event was assessed using a 5-point scale that captures severity and the degree to which activities of daily living (ADL) are affected:

- Grade 1: Asymptomatic or mild symptoms; only clinical or diagnostic findings (motor neuropathy); asymptomatic (sensory neuropathy).
- Grade 2: Moderate symptoms limiting instrumental ADL (e.g., meal preparation, shopping, managing finances).
- Grade 3: Severe symptoms limiting self-care ADL (e.g., bathing, dressing, feeding).
- Grade 4: Life-threatening consequences requiring urgent intervention.
- Grade 5: Death.

The time until the development of grade 2 or higher peripheral neuropathy was assessed over an 8-week timeframe, measured as the duration in weeks from the initial paclitaxel administration.

##### 2.4.1.1 Patient quality of life (QoL)

(QoL) was measured using the Arabic-adapted version of the Functional Assessment of Cancer Therapy/Gynecologic Oncology Group–Neurotoxicity (FACT-GOG-Ntx) subscale (Calhoun et al., 2003). This validated instrument comprises 11 items addressing neuropathy symptoms. For each item, patients responded on a 5-point Likert scale (0–4), and responses were recorded item by item. Item scores within each subscale were summed according to the FACT-GOG-Ntx scoring guidelines (including any required item reversals), and subscale scores were then added to obtain the total FACT-GOG-Ntx score. Because there were no missing items, no prorating was required. The total score ranged from 0 to 44; lower values correspond to greater symptom burden. A minimal clinically important difference (MCID) was established as a worsening greater than 10% from baseline FACT-Ntx scores, a threshold considered indicative of clinically relevant neuropathy progression. The incidence of MCID was compared between treatment arms at study completion (Haroun et al., 2023; Cella et al., 1993).

#### 2.4.2 Secondary outcomes

##### 2.4.2.1 Pain severity assessment

Neuropathic pain severity was evaluated at rest at weeks 4 and 8 using the Visual Analog Scale (VAS), categorizing pain intensity as: 0 (no pain), 1–3 (mild), 4–6 (moderate), and 7–10 (severe). This standardized approach facilitated systematic assessment of pain progression and treatment effects (Delgado et al., 2018).

#### 2.4.3 Exploratory endpoints

Serum samples were collected at both baseline and the end of the study to assess the neuropathy biomarker, nerve growth factor (NGF). Approximately 8 mL of venous blood was drawn using sterile venipuncture under strict aseptic conditions. The sample was divided into portions: 2 mL was placed in an EDTA tube for measuring total leukocyte count (TLC), lymphocyte count, and platelet count; 3 mL was transferred into a plain tube for assessing aspartate aminotransferase (AST), alanine aminotransferase (ALT), urea, and creatinine levels. The remaining 3 mL was allowed to clot, then centrifuged, and the serum was collected and stored at −20 °C until NGF measurement. TLC, lymphocytes, and platelet counts were determined using an automated cell counter (CelltacES, Nihon Kohden, Germany). AST and ALT levels were analyzed with a fully automated chemistry analyzer (Selectra Pro XL 16–8,361, Eli Tech Group Clinical Systems, Germany). Finally, nerve growth factor was measured via enzyme-linked immunosorbent assay (ELISA) (Human Nerve growth factor, NGF, Catalog No: E2102Hu, Bioassay Technology Laboratory, China) following manufacturer protocols. The percentage change in biomarker levels was calculated as:

$$\text{Percentage Change }\left(\%\right)=\left(\text{Biomarker }{\text{Level}}_{\text{End}}-\text{Biomarker }{\text{Level}}_{\text{Baseline}}\;/\text{ Biomarker }{\text{Level}}_{\text{Baseline}}\right)\times 100$$

where ${\text{Biomarker}\;\text{Level}}_{\text{Baseline}}$ represents the biomarker concentration measured before treatment, and ${\text{Biomarker}\;\text{Level}}_{\text{End}}$ represents the measurement taken at the conclusion of the intervention duration.

##### 2.4.3.1 Patient assessment and follow-up

Initial assessments were performed 1 week before paclitaxel initiation to allow stabilization before randomization. Baseline demographic, laboratory, and clinical information were gathered via patient interviews and medical records.

Peripheral neuropathy incidence was systematically evaluated during each paclitaxel cycle. Patient-reported outcomes, including QoL and pain severity, were assessed twice during the study period—midpoint (week 4) and completion (week 8)—chosen to capture the dose-dependent pattern of neuropathy onset and escalation. All clinical and patient-reported assessments were performed 1–2 h prior to paclitaxel infusion to ensure consistency. Biomarker measurements for NGF were obtained at baseline and after the eighth cycle to explore underlying biochemical treatment effects.

### 2.5 Safety monitoring

Patient safety was monitored weekly during chemotherapy cycles through face-to-face visits focusing on treatment adherence and adverse events documentation. Adherence was tracked using prescription refills and pill counts, with patients below 90% adherence deemed non-compliant and excluded from analysis. Adverse events were carefully recorded from patient reporting, laboratory data, and medical records to maintain a detailed safety profile.

### 2.6 Statistical analysis

#### 2.6.1 Power and sample size estimation for two-proportion tests

Calculation of sample size was conducted using the SampSize App Version 3. Assuming two independent groups with a dichotomous primary endpoint (incidence of NCI-CTCAE grade ≥2 peripheral neuropathy) and given the novelty of investigating trimetazidine’s effect on paclitaxel-induced neuropathy, no prior effect size data were available. An effect size of 0.46 was assumed, with a two-sided alpha of 0.05% and 90% power, assuming equal allocation between groups. This yielded a required sample size of 30 patients per arm, which was increased to 35 per arm to accommodate an expected dropout rate of 15%. Consequently, A total of seventy individuals were enrolled in the current study.

#### 2.6.2 Statistical methodology

Data analysis was conducted using IBM© SPSS© software version 25 (IBM© Corp., Armonk, NY, United States). The Kolmogorov-Smirnov test evaluated data normality. Quantitative variables with normal distribution are reported as mean ± standard deviation (SD), while non-normally distributed data are summarized using median and interquartile range (IQR). Categorical variables are presented as frequencies and percentages. Group comparisons utilized the Independent Samples t-test for normally distributed data and the Mann-Whitney U test for non-parametric data. Chi-square or Fisher’s exact test was applied for categorical variables. The Wilcoxon signed-rank test was used for paired non-parametric data. Kaplan-Meier analysis was employed for survival outcomes. Statistical significance was defined as p < 0.05.

## 3 Results

**FIGURE 1:**
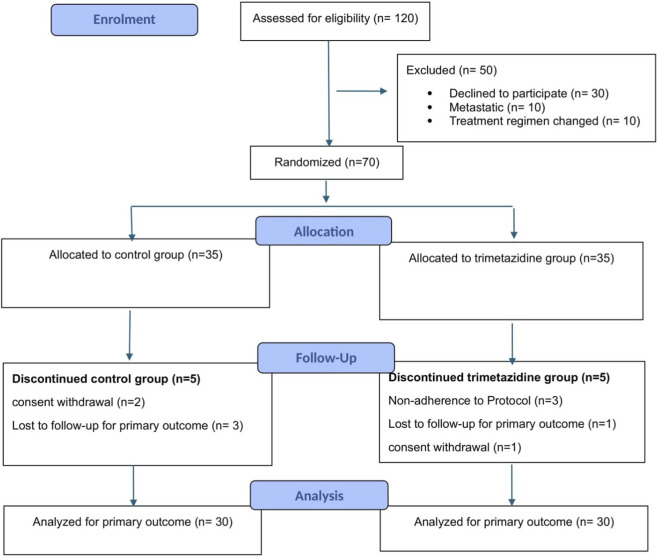
CONSORT 2025 flow diagram.

Between June 2024 and January 2025, 120 patients were screened, with 70 meeting eligibility criteria and randomized equally to trimetazidine (n = 35) or placebo (n = 35) groups. Ten patients discontinued due to non-compliance with the trimetazidine dosing regimen (n = 3), consent withdrawal (n = 3), or loss to follow-up (n = 4). Sixty patients completed the study and were included in the final analysis. Participant flow is illustrated in Figure 1.

### 3.1 Patient’s demographics and baseline clinical data

Participant baseline demographic and clinical features are summarized in Table 1. The mean age was 46.9 ± 9.6 years in the trimetazidine group and 46.9 ± 10.1 years in the control group (p = 0.979). Both groups demonstrated comparable body surface area measurements, averaging 1.7 ± 0.1 m^2^. Clinical parameters included relevant laboratory values and tumor characteristics. Overall, no significant differences were observed between the groups across these baseline variables, indicating well-balanced cohorts that support the integrity and comparability of subsequent analyses.

**TABLE 1:** Baseline demographic and clinical characteristics of study participants.

Variable		Group		P value
		Control	Trimetazidine	
		N = 30	N = 30	
Age (year) Mean ± SD		46.9 ± 10.1	46.9 ± 9.6	0.979^a^
BSA (m^2^) Mean ± SD		1.7 ± 0.1	1.7 ± 0.1	0.922^a^
Educational level, n (%)	Illiterate < 12 years educ. > 12 years educ.	7 (23.3%) 11 (36.7%) 12 (40%)	7 (23.3%) 8 (26.7%) 15 (50%)	0.668^b^
Menopausal status, n (%)	Premenopausal Postmenopausal	19 (63.3%) 11 (36.7%)	19 (63.3%) 11 (36.7%)	1^b^
Comorbidities, n (%)	None HTN Dyslipidemia NFALD	24 (80%) 5 (16.7%) 0 (0%) 1 (3.3%)	25 (83.3%) 4 (13.3%) 1 (3.3%) 0 (0%)	0.546^b^
Medication profile, n (%)	None ARBs B-blockers CCB Statins	24 (80%) 1 (3.3%) 3 (10%) 1 (3.3%) 1 (3.3%)	25 (83.3%) 0 (0%) 3 (10%) 1 (3.3%) 1 (3.3%)	0.907^b^
ECOG, n (%)	0 1	22 (73.3%) 8 (26.7%)	23 (76.7%) 7 (23.3%)	0.766^b^
Estrogen receptor, n (%)	Negative Positive	7 (23.3%) 23 (76.7%)	11 (36.7%) 19 (63.3%)	0.260^b^
Progesterone receptor, n (%)	Negative Positive	6 (20%) 24 (80%)	11 (36.7%) 19 (63.3%)	0.152^b^
HER2, n (%)	Negative Positive Equivocal	18 (60%) 7 (23.3%) 5 (16.7%)	17 (56.7%) 9 (30%) 4 (13.3%)	0.823^b^
Lymph node, n (%)	N0 N1 N2 N3	7 (23.3%) 10 (33.3%) 9 (30%) 4 (13.3%)	9 (30%) 7 (23.3%) 10 (33.3%) 4 (13.3%)	0.842^b^
Tumor size, n (%)	T1 T2 T3	8 (26.7%) 13 (43.3%) 9 (30%)	2 (6.7%) 17 (56.7%) 11 (36.7%)	0.115^b^
Tumor stage, n (%)	Stage I Stage II Stage III	7 (23.3%) 12 (40%) 11 (36.7%)	2 (6.7%) 19 (63.3%) 9 (30%)	0.102^b^
Aspartate aminotransferase (U/L) Mean ± SD		30.3 ± 8.3	27.3 ± 8.9	0.182^a^
Alanine aminotransferase (U/L) Mean ± SD		25 ± 8.5	25.1 ± 8.8	0.976^a^
Serum creatinine (mg/dL) Mean ± SD		0.6 ± 0.2	0.7 ± 0.2	0.125^a^
Urea (mg/dL) Mean ± SD		18.1 ± 2.4	17.8 ± 2.7	0.653^a^
Platelet count (×10^9^/L) Mean ± SD		293.2 ± 80.8	292.1 ± 81.1	0.957^a^
Lymphocyte count (×10^9^/L) Mean ± SD		3.2 ± 1.1	2.9 ± 1.1	0.379^a^
Total leukocyte count (TLC) (×10^9^/L) Mean ± SD		6.8 ± 2.37	6.55 ± 2.64	0.708^a^
Cumulative dose of chemotherapy, Mean ± SD	Doxorubicin (mg)	408.2 ± 32.8	407.4 ± 30.4	0.922^a^
	Cyclophosphamide (mg)	4,081.6 ± 327.6	4,073.6 ± 303.6	0.922^a^
	Paclitaxel (mg)	1,088.4 ± 87.3	1,086.3 ± 81	0.922^a^

Abbreviations: BSA, body surface area; HTN, hypertension; NFALD, nonalcoholic fatty liver disease; ARBs, angiotensin II, receptor blockers; CCB, calcium channel blocker; ECOG, eastern cooperative oncology group; HER2, Human epidermal growth receptor 2; n, number of patients.

^a^ Independent Samples T-test for quantitative data between the two groups.

^b^ Chi square test for qualitative data between the two groups.

Significant level at P value < 0.05.

### 3.2 Incidence of peripheral neuropathy

Neuropathy grading according to NCI- CTCAE version 5.0. were recorded after each cycle of paclitaxel. At the end of 8th cycle, for peripheral sensory neuropathy, the trimetazidine group showed a lower proportion of patients with Grade 2 or 3 events (63.3%) versus 80% in the control group, although this difference was not statistically significant (p = 0.152). Significant reductions were seen with trimetazidine in other symptoms: paresthesia Grade 2 or 3 was significantly lower (63.3% vs. 86.7%, p = 0.037), as was peripheral motor neuropathy Grade 2 or 3 (33.3% vs. 70%, p = 0.004), and dysesthesia Grade 2 or 3 (60% vs. 83.3%, p = 0.045). No participants in the study experienced neuropathy of grade 4 or higher. The incidence and grading details are presented in Table 2.

**TABLE 2:** Incidence of developing NCI- CTCAE grade 2 or 3 Neuropathy.

Moderate to severe events		Group		P value
		Control	Trimetazidine	
		N = 30	N = 30	
Peripheral sensory neuropathy	Grade 1 Grade 2 or 3	6 (20%) 24 (80%)	11 (36.7%) 19 (63.3%)	0.152
Paresthesia	Grade 1 Grade 2 or 3	4 (13.3%) 26 (86.7%)	11 (36.7%) 19 (63.3%)	0.037*
Peripheral motor neuropathy	Grade 1 Grade 2 or 3	9 (30%) 21 (70%)	20 (66.7%) 10 (33.3%)	0.004*
Dysesthesia	Grade 1 Grade 2 or 3	5 (16.7%) 25 (83.3%)	12 (40%) 18 (60%)	0.045*

Chi square test for qualitative data between the two groups.

*: Significant level at P value < 0.05.

### 3.3 Time to develop peripheral neuropathy

**FIGURE 2:**
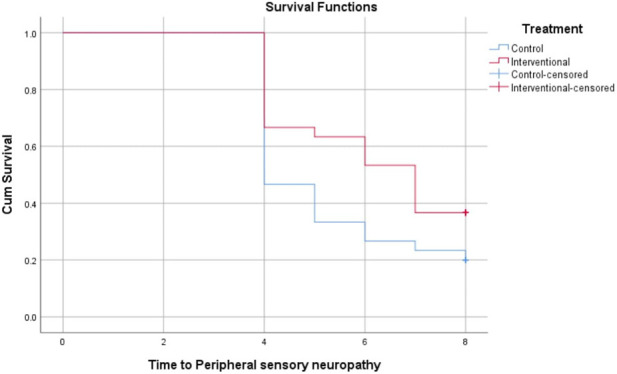
Kaplan-Meier Curve for time to development of grade 2 or 3 peripheral sensory neuropathy.

**FIGURE 3:**
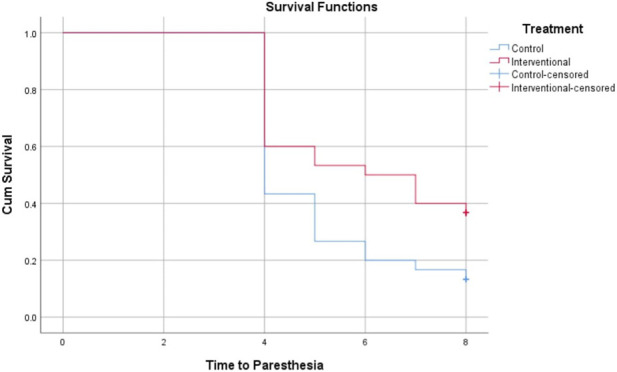
Kaplan-Meier Curve for time to development of grade 2 or 3 paresthesia.

**FIGURE 4:**
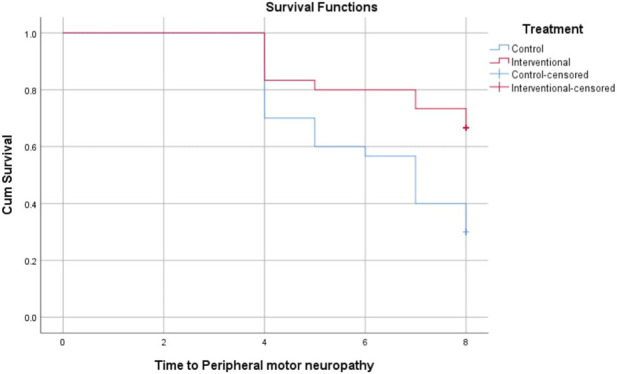
Kaplan-Meier Curve for time to development of grade 2 or 3 peripheral motor neuropathy.

**FIGURE 5:**
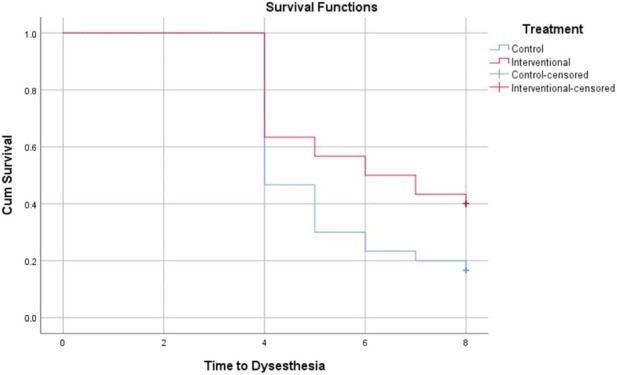
Kaplan-Meier Curve for time to development of grade 2 or 3 dysphasia.

The Kaplan-Meier analysis in Table 3 evaluates the time in weeks till the onset of grade 2 or 3 peripheral neuropathy across different neuropathy types between the control and intervention groups. For peripheral sensory neuropathy, the intervention group showed a longer mean time to onset (6.2 weeks) compared to the control (5.3 weeks), with borderline statistical significance (P = 0.069). Significant delays in onset were observed for paresthesia (6.0 vs. 5.1 weeks, P = 0.026), peripheral motor neuropathy (7.2 vs. 6.3 weeks, P = 0.005), and dysesthesia (6.1 vs. 5.2 weeks, P = 0.035) favoring the intervention. These findings suggest that the intervention effectively prolongs the time before the development of clinically meaningful peripheral neuropathy symptoms in the patient population. Data are represented in Figures 2–5.

**TABLE 3:** Kaplan-Meier estimated time (Weeks) to development of grade 2 or 3 peripheral neuropathy by neuropathy type and treatment group.

Neuropathy type	Group	Estimate mean	95% confidence interval		P value log rank (mantel-cox)
			Lower bound	Upper bound	
Peripheral sensory neuropathy	Control	5.3	4.7	5.9	0.069
	Intervention	6.2	5.6	6.8	
Paresthesia	Control	5.1	4.5	5.6	0.026*
	Intervention	6	5.4	6.7	
Peripheral motor neuropathy	Control	6.3	5.6	6.9	0.005*
	Intervention	7.2	6.6	7.7	
Dysesthesia	Control	5.2	4.6	5.8	0.035*
	Intervention	6.1	5.5	6.8	

*: Significant level at P value < 0.05.

### 3.4 Quality of life

Table 4 presents the FACT-GOG-Ntx subscale scores comparing the control and trimetazidine groups at baseline, Week 4, and Week 8. At baseline, both groups had similar median scores, indicating comparable neuropathy-related quality of life before intervention (median scores: control 39 vs. trimetazidine 41, P = 0.304). However, at both Week 4 and Week 8, the trimetazidine group showed significantly higher median scores, reflecting better preservation of quality of life (Week 4: 35 vs. 29, P < 0.001; Week 8: 34 vs. 24, P < 0.001). Both groups demonstrated significant within-group score reductions over time (P < 0.001). Additionally, the proportion of patients achieving a minimal clinically important difference (MCID) was significantly higher in the control group (96.6%) compared to the trimetazidine group (73.33%, P = 0.026), indicating fewer clinically meaningful declines in the intervention arm. These results suggest that trimetazidine may mitigate the deterioration in peripheral neuropathy-related quality of life over the treatment period.

**TABLE 4:** Fact- GOG- Ntx subscale scores of the two study arms.

Ntx subscale score		Group		P value
		Control	Trimetazidine	
		N = 30	N = 30	
Baseline	Median (IQR)	39 (37.3–42)	41 (38–43)	0.304 ^a^
At the end of week 4	Median (IQR)	29 (26–30.8)	35 (34–35)	<0.001^a^ *
At the end of week 8	Median (IQR)	24 (21–25.8)	34 (33–34)	<0.001^a^*
P Value		<0.001^b^*	<0.001^b^*	​
MCID N%	​	29 (96.6%)	22 (73.33%)	0.026^c^*

Abbreviation: MCID, minimal clinically important difference.

^a^ Mann Whiteney for quantitative data between the two groups.

^b^ Freidman test for quantitative data between two times within each group.

^c^ Fishers Exact Test for qualitative data between the two groups.

*: Significant level at P value < 0.05.

### 3.5 Severity of pain

Table 5 summarizes pain severity distribution between the control and trimetazidine groups at Week 4 and Week 8. By Week 4, a significantly greater percentage of patients in the trimetazidine group experienced either no pain or mild pain compared to the control group (P = 0.020). By Week 8, this difference persisted, with more patients in the trimetazidine arm experiencing no or mild pain versus control (P = 0.015). Within-group comparisons showed significant changes in pain severity between Week 4 and Week 8 for both groups (control, P < 0.001; trimetazidine, P = 0.014), indicating pain progression over time. These data suggest trimetazidine may contribute to reduced pain severity during treatment.

**TABLE 5:** Pain Severity Assessment at Week 4 and Week 8 in Control and trimetazidine Groups.

Pain severity		Group		P value
		Control	Trimetazidine	
		N = 30	N = 30	
At the end of week 4	No pain Mild Moderate Severe	4 (13.3%) 10 (33.3%) 7 (23.3%) 9 (30%)	13 (43.3%) 7 (23.3%) 8 (26.7%) 2 (6.7%)	0.020 ^a^*
At the end of week 8	No pain Mild Moderate Severe	3 (10%) 5 (16.7%) 7 (23.3%) 15 (50%)	12 (40%) 6 (20%) 7 (23.3%) 5 (16.7%)	0.015 ^a^*
P Value (4W vs. 8W)		<0.001 ^b^*	0.014 ^b^*	​

^a^ Chi square test for qualitative data between the two groups.

^b^ Wilcoxon Signed rank test for quantitative data between two times within each group.

*: Significant level at P value < 0.05.

### 3.6 Serum biomarkers

**FIGURE 6:**
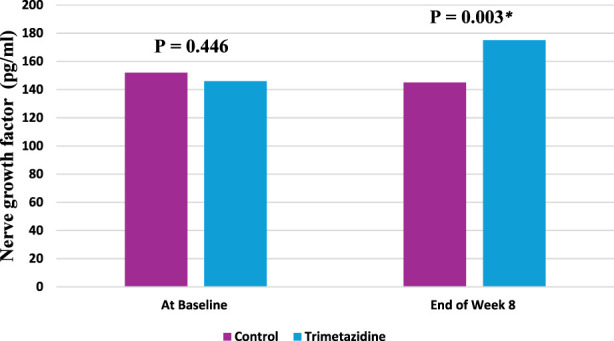
Nerve Growth Factor (NGF) Median Levels at Baseline and Week 8 in Control and Trimetazidine Groups. *: Significant level at P value <0.05.

Figure 6 presents the median nerve growth factor (NGF) levels (pg/mL) in both control and trimetazidine groups at baseline and Week 8, along with the corresponding interquartile ranges (IQR) reported in the text. At baseline, NGF levels were comparable between groups (control: 152 [136–172], trimetazidine: 146 [133–169]; P = 0.446). By Week 8, patients treated with trimetazidine exhibited a significant elevation in NGF levels compared to the control group (175 [143–205] vs. 145 [123–159]; P = 0.003). Furthermore, the median percentage change in NGF was markedly higher in the trimetazidine group (25.9) compared to a decrease in controls (−5.54; P < 0.001). These data indicate a significant elevation of NGF levels in the intervention arm over the study period.

### 3.7 Safety

The trimetazidine safety profile was analogous to that observed in the control cohort (all P values >0.05), as delineated in Table 6. Adverse events reported across both arms encompassed anemia, neutropenia, alopecia, diarrhea, fatigue, hypersensitivity reactions, dizziness, and headache. These events were uniformly mild and self-resolving, requiring no targeted therapeutic intervention, aside from two instances of grade 3 anemia in the control group, which necessitated blood transfusion. Crucially, no episodes of significant hemorrhage were documented. Furthermore, the frequency and severity of prevalent paclitaxel-associated toxicities were similar between the two groups without statistically significant differences.

**TABLE 6:** Incidence and Severity of Other Adverse Events During Treatment in Control and trimetazidine Groups.

Adverse event, n (%)		Group		P value
		Control	Trimetazidine	
		N = 30	N = 30	
Anemia	None Grade 1 Grade 2 Grade 3	13 (43.3%) 10 (33.3%) 5 (16.7%) 2 (6.7%)	10 (33.3%) 16 (53.3%) 4 (13.3%) 0 (0%)	0.274
Neutropenia	None Grade 1 Grade 2	16 (53.3%) 10 (33.3%) 4 (13.3%)	13 (43.3%) 14 (46.7%) 3 (10%)	0.571
Alopecia	None Grade 1	2 (6.7%) 28 (93.3%)	1 (3.3%) 29 (96.7%)	0.554
Diarrhea	None Grade 1 Grade 2	16 (53.3%) 9 (30%) 5 (16.7%)	16 (53.3%) 10 (33.3%) 4 (13.3%)	0.921
Fatigue	None Grade 1 Grade 2	7 (23.3%) 21 (70%) 2 (6.7%)	7 (23.3%) 20 (66.7%) 3 (10%)	0.894
Allergic reaction	None Grade 1 Grade 2	27 (90%) 3 (10%) 0 (0%)	25 (83.3%) 4 (13.3%) 1 (3.3%)	0.543
Dizziness	None Grade 1 Grade 2	27 (90%) 2 (6.7%) 1 (3.3%)	26 (86.7%) 3 (10%) 1 (3.3%)	0.896
Headache	None Grade 1 Grade 2	26 (86.7%) 2 (6.7%) 2 (6.7%)	24 (80%) 4 (13.3%) 2 (6.7%)	0.688

Grade 1 anemia (hemoglobin < Lower Limit of Normal – 10.0 g/dL); grade 2 (hemoglobin <10.0–8.0 g/dL); grade 3 (hemoglobin <8.0 g/dL, transfusion indicated). Grade 1 neutropenia (neutrophil count: <2.0–1.5 × 10^9^/L); grade 2 (neutrophil count: <1.5–1.0 × 10^9^/L). Grade 1 Alopecia (Hair loss of <50% of normal for that individual that is not obvious from a distance but only on close inspection). Grade 1 diarrhea (Increase of <4 stools per day over baseline); grade 2 (Increase of 4 - 6 stools per day over baseline). Grade 1 Fatigue (Fatigue relieved by rest); grade 2 (Fatigue not relieved by rest; limiting instrumental activities of daily living). Grade 1 Allergic reaction (Systemic intervention not indicated); grade 2 (Oral intervention indicated). Grade 1 Dizziness (Mild unsteadiness or sensation of movement); grade 2 (Moderate unsteadiness or sensation of movement; limiting instrumental activities of daily living). Grade 1 headache (mild pain); grade 2 (Moderate pain; limiting activities of daily living).

Chi square test for qualitative data between the two groups.

Significant level at P value < 0.05.

## 4 Discussion

Trimetazidine confers neuroprotection in peripheral neuropathy chiefly by modulating mitochondrial bioenergetics and reducing oxidative stress and inflammation, notably via the TLR4/NF-κB pathway. These mechanisms help preserve neuronal health and reduce neuropathic symptoms. By optimizing cellular metabolism and suppressing proinflammatory signals, trimetazidine shows promise in ameliorating chemotherapy-induced neurotoxicity (Elbaset et al., 2024; Hammad A. et al., 2023).

This study is the first clinical trial assessing trimetazidine’s potential protective effect against paclitaxel-induced peripheral neuropathy. The primary outcome was the difference in incidence of grade 2 or 3 neuropathy between groups, evaluated via the NCI-CTCAE criteria (Griffith et al., 2014), encompassing four distinct manifestations of peripheral neuropathy—motor, sensory, paresthesia, and dysesthesia reflecting different neuropathic patterns. The validated NCI-CTCAE system ensures consistent and reproducible patient-reported assessments and is commonly used in trials on chemotherapy-induced neuropathy (Haroun et al., 2023; Khalefa et al., 2020).

Our results showed that trimetazidine treatment significantly reduced the incidence of grade 2 or 3 neuropathic symptoms in patients on paclitaxel chemotherapy, notably decreasing paresthesia (p = 0.037), peripheral motor neuropathy (p = 0.004), and dysesthesia (p = 0.045). The reduction in peripheral sensory neuropathy was not statistically significant (p = 0.152), likely due to variability in sensory symptom reporting, patient perception differences, and sample size limitations. Sensory neuropathy’s heterogeneous nerve involvement may also dilute group differences. Kaplan-Meier analysis revealed that trimetazidine significantly delayed the onset of neuropathic symptoms. Specifically, sensory neuropathy onset was delayed by approximately 0.9 weeks in the trimetazidine group (6.2 vs. 5.3 weeks; p = 0.069), with more pronounced delays for paresthesia (6.0 vs. 5.1 weeks; p = 0.026), peripheral motor neuropathy (7.2 vs. 6.3 weeks; p = 0.005), and dysesthesia (6.1 vs. 5.2 weeks; p = 0.035).

Considering the lack of similar clinical trials, trimetazidine’s neuroprotective effect observed in this study is supported by extensive preclinical evidence. Animal models consistently show trimetazidine’s multifaceted protection in peripheral neuropathy. Elbaset et al. (2024) demonstrated its mitigation of cisplatin-induced neuropathy via modulation of AMPK-mediated PI3K/mTOR, Nrf2, and NF-κB signaling, reducing neuro-oxidative stress and inflammation while preserving nerve structure and function. Hazem et al. (2023), reported that trimetazidine alleviates paclitaxel-induced neuropathy by targeting TLR4/p38/NF-κB signaling and enhancing klotho protein expression, underscoring its anti-inflammatory and neuroprotective actions. Gölboyu et al. (2023) found that trimetazidine protects against diabetic polyneuropathy by modulating soluble HMGB1, a proinflammatory mediator linked to nerve damage. Additionally, trimetazidine impacts tumor metabolism by inhibiting glycolysis and AKT pathways and upregulating neuronal progranulin with ERK1/2 signaling modulation, contributing to antinociceptive effects in chemotherapy neuropathy (Nasser et al., 2022; Hazem et al., 2023).

Quality of life was assessed using the FACT-NTx subscale, a validated and reliable tool commonly used in oncology to measure the effects of chemotherapy-induced neuropathy. Both groups showed significant declines in FACT-NTx scores from baseline to the end of treatment (p < 0.001), reflecting CIPN’s negative impact on quality of life, consistent with prior studies in breast cancer patients receiving taxanes (Schwab and Visovsky, 2023; Salehifar et al., 2020). Trimetazidine use was linked to fewer patients experiencing clinically significant declines in quality of life (73.33% vs. 96.6%, p = 0.026). Pain severity, measured by VAS, was consistently lower in the trimetazidine group at weeks 4 and 8, compared to the control group, although all patients reported increased pain relative to baseline, aligning with findings by Takemoto et al. (2012). This suggests that trimetazidine may attenuate neuropathic pain severity during chemotherapy.

Nerve Growth Factor (NGF) plays a key role in neuropathic pain by modulating nociceptor sensitization, neuronal plasticity, and inflammatory pathways. Although NGF is crucial biologically, its reliability as a therapeutic biomarker requires further validation due to variability in clinical findings and the need for standardization. NGF-targeted therapies show promise, but more research is needed to establish their clinical utility (Youk et al., 2017; Cavaletti et al., 2004; Soejitno and Widyadharma, 2024).

The restorative effect of trimetazidine, suggested by the greater median percentage increase in NGF within the treatment group (25.9%) compared to controls (−5.54%), aligns with the findings of Haroun et al. (2023) and supports the notion that NGF acts as a crucial neurotrophic factor whose restoration can attenuate neuropathic symptoms. This interpretation is reinforced by evidence linking CIPN development to NGF depletion, a mechanism strongly supported by the prospective study by Youk et al. (2017), which demonstrated that in patients receiving certain chemotherapies (e.g., bortezomib, thalidomide), the development of CIPN was associated with a significant drop in NGF levels from baseline (−3.52 ± 5.72 pg/mL). This concept of neurotrophic deficit is further reinforced by the cross-sectional study by Irsyad et al. (2025), which found that the median serum NGF level was significantly lower in cancer patients with established CIPN (103.26 pg/mL) compared to those without CIPN (148.91 pg/mL).

Conversely, the association reported by Velasco et al. (2017) of elevated serum NGF with increased neuropathic pain suggests a distinct, pronociceptive role, where NGF sensitizes pain fibers. Taken together, this body of evidence suggests a duality: while a decrease in NGF may be indicative of structural damage and neurotrophic factor deprivation (supporting a restorative therapeutic approach), an increase may signify neuronal sensitization and pain signaling rather than a definitive neuroprotective effect. These discrepancies emphasize the need for future studies to stratify CIPN based on both the causative agent and the predominant symptom (pain vs. functional deficit) to accurately define NGF’s utility as a prognostic biomarker and therapeutic target.

Trimetazidine was well tolerated and did not increase paclitaxel toxicity. The large ATPCI trial (Dalal and Kapoor, 2021) with 6,007 patients showed no significant difference in serious adverse events between trimetazidine (40.9%) and placebo (41.1%) over a median 47.5 months (HR 0.98; 95% CI 0.88–1.09; p = 0.73). Meiszterics et al. (2017) reported only 1.1% mild, reversible treatment-related adverse events during 6 months of therapy with no deaths. These findings confirm trimetazidine’s safety and absence of added chemotherapy toxicity, consistent with our study results.

The study has several potential limitations. First, the final sample size of 60 patients, although meeting the a priori per-arm requirement, may still limit statistical power and the ability to detect modest between-group differences in neuropathy outcomes, and therefore the findings should be interpreted as exploratory and hypothesis-generating rather than definitive. Second, the 8-week duration of paclitaxel treatment may be insufficient to capture long-term neuropathy outcomes, delayed ‘coasting’ phenomena, or late onset effects, and longer post-treatment follow-up are needed to fully characterize these aspects. Third, the use of subjective scales such as NCI-CTC and FACT-GOG-NTx, and VAS while validated, may introduce some measurement bias. Finally, the use of a fixed dose of 35 mg trimetazidine, although supported by previous studies, may not allow exploration of dose-response relationships or optimization of therapeutic effects. Additionally, the standardization and validation of NGF as a clinical biomarker for CIPN remain limited, which may affect interpretation of biomarker findings.

## 5 Conclusion

In summary, trimetazidine effectively reduced the incidence of PIPN during the early phase of treatment, specifically within the first 8 weeks. This neuroprotective benefit was mirrored by improvements in patients’ quality of life. Its favorable safety and tolerability profile underline the potential clinical value of trimetazidine. Nonetheless, larger trials with extended follow-up and stratified patient groups are essential to validate these findings further.

## Data Availability

The raw data supporting the conclusions of this article will be made available by the authors, without undue reservation.
